# Human–computer interaction tools with gameful design for critical thinking the media ecosystem: a classification framework

**DOI:** 10.1007/s00146-022-01583-z

**Published:** 2022-11-01

**Authors:** Elena Musi, Lorenzo Federico, Gianni Riotta

**Affiliations:** 1 Department of Communication and Media, University of Liverpool, 19 Abercromby Square, Liverpool, L69 7ZG UK; 2 Datalab, University Luiss Guido Carli, Viale Pola 12, Rome, Italy

**Keywords:** Fake news, Human–computer interaction, Critical thinking, Data literacy

## Abstract

In response to the ever-increasing spread of online disinformation and misinformation, several human–computer interaction tools to enhance data literacy have been developed. Among them, many employ elements of gamification to increase user engagement and reach out to a broader audience. However, there are no systematic criteria to analyze their relevance and impact for building fake news resilience, partly due to the lack of a common understanding of data literacy. In this paper we put forward an operationalizable definition of data literacy as a form of multidimensional critical thinking. We then survey 22 existing tools and classify them according to a framework of 10 criteria pointing to their gameful design and educational features. Through a comparative/contrastive analysis informed by a focus group, we provide a principled set of guidelines to develop more efficient human–computer interaction tools to teach how to critically think in the current media ecosystem.

## Introduction

The infodemic has shown us that public ability to recognize false and misleading information during a disease outbreak is crucial to diminishing (a) risk-taking behaviors by the misinformed and (b) mistrust in institutions and media which hampers public health responses and recovery. Due to the proliferation of information across digital media, fact-checking is struggling to keep up with the spread of misinformation. As a result, the number of infodemically vulnerable people is increasing at a rapid pace (https://reutersinstitute.politics.ox.ac.uk/UK-COVID-19-news-and-information-project).

The phenomenon of misinformation has been exacerbated by the advent of Networked Society and the rise of AI systems designed to accomplish tasks mimicking how the brain works rather than helping humans evaluate their reasoning patterns. As a result, while advances in natural language generation leveraging GPT-3 produce systems able to produce news articles not distinguishable from those written by journalists (https://www.theguardian.com/commentisfree/2020/sep/08/robot-wrote-this-article-gpt-3, automatic fact-checking systems still struggle to identify disinformation bundles.

To counter this scenario, the European Commission advocated already 15 years ago (2007) for a media literacy campaign targeting mediatic systems in the digital infosphere, including their economic and cultural dimensions. Such urgency was reiterated in *the Digital Competence Framework for Citizens* proposed by “The Digital Education Action Plan” (2018) with a stress on *data literacy*. Skills designated under this umbrella term are received differently by different demographics and constitute a challenge for standard curricula due to fast-pace changes.

Gamification environments based on human–computer interaction have proved to be an efficient learning tool since, while boosting users’ digital skills, they provide rapid feedback, they guarantee freedom to fail, a sense of progression and a storytelling environment that prompts focus and concentration (Stott and Neustaedter 2013). A variety of media literacy games hosted on digital platforms are currently publicly accessible. As underlined by Miles and Lyons’ cross-indexing (2019), these games differ in terms of learning outcomes, type of learning experience (e.g., knowledge anticipation vs. reflection) as well as type of action (e.g., simulation or puzzle). However, there is currently no systematic framework to assess which data literacy skills are addressed and to evaluate systems’ design in view of their desired outcome. This is partially due to the lack of an agreed notion of *data literacy*, which remains a blurred concept to be updated hand in hand with changes in the digital media ecosystem. More specifically, as pointed out by Carmi et al. (2020), there is confusion among scholars and policy makers about what *critical thinking* means when applied to the *digital ecosystem*. As a result, existing human–computer interaction tools are addressing only some aspects of critical thinking skills for media literacy in the online (mis)information ecosystem. To inform the future development of such educational tools, we propose a classification framework to analyze existing tools in a functional perspective. The paper is organized as follows: Sect. 2 discusses the underpinnings of critical thinking for media literacy in relation to the misinformation ecosystem within the digitized society, identifying five components. Drawing from the operationalizable definition of critical thinking for media literacy and from the literature review, Sect. 3.1 puts forward a suite of 10 criteria to classify human–computer interaction tools to teach media literacy. Section 3.2 reports a survey of existing tools analyzed against the criteria with the aid of a focus group. On the backdrop of the survey results, Sect. 4 is devoted to the discussion of limitations of currently available tools. Section 5 summarizes the theoretical and empirical contributions of the study, offering recommendations to inform the design of a new generation of human–computer interaction tools to teach critical thinking for media literacy.^1^

## Critical thinking and data literacy in the Networked Society

The relevance of critical thinking for media literacy has been pointed out before the datafication of the infosphere. Koltay (2011) lists “Having a critical approach to quality and accuracy of content” as the second level of the five making up media literacy. However, in the era of echo-chambers and filter bubbles, it sounds intuitive that quality and accuracy of content are tightly bound to digital platforms’ affordances and infrastructure. Such an awareness cannot be taken for granted: according to the last *Digital Understanding Report* (2018) by Doteveryone, almost two-thirds of people (62%) do not realize that the news they see online highly depends on their social media. On these grounds, the NGO mentions “thinking critically about the trustworthiness of information” as one of the pillars to make citizens aware of the public sphere. In the attempt to decipher the underpinnings of *critical thinking* the online media ecosystem, Carmi et al. (2020) suggest reframing “critical thinking” as “data thinking”, critical understanding of data collection, data economy and, more in general, data cycle.

If we adopt the traditional definition of critical thinking as “active, persistent and careful consideration of any belief or supposed form of knowledge in light of the grounds that support it, and the further conclusions to which it tends” (Dewey 1910: 6; 1933: 9), it is apparent that the dimensions of content and data are closely intertwined in the information ecosystem: the arguments that support a news claim ground it as much as its algorithmically-induced popularity. The situation is further complicated by the presence of different types of information disorders which include misinformation—information that is misleading, but not necessarily containing non-factual information—and disinformation—nonfactual information created with the intention of deceiving. As remarked by Nobel Prize Maria Ressa in her last interview at the Oxford internet Institute, the digital has reshaped the backbones of the news-making process: “the biggest change that has happened is that we used to be the gatekeepers. Journalists were trained to make decisions about who was right and who was wrong. Who gets the megaphone, and who does not… All of a sudden, social media platforms have been given that power” while, at the same time, you are intimately connected to your community in real time (https://tinyurl.com/atj44vhf). In other words, the news-making process entails a negotiation of opinions in argumentative polylogical discussions sparse on the web (Musi and Aakhus 2018): multiple players exchange positions within and across a variety of digital places. News making has always been both a rhetorical and argumentative exercise since aimed at gaining the acceptance of a certain interpretation of a news event. Nevertheless, digitization has brought structural changes in the dynamics of information exchange that touch upon every of the **Five Ws:**

- **who**: digital media platforms have opened up the era of citizens’ journalism (Glaser 2006) allowing anyone to create, edit and share news. This enlarged participation can increase the risk of low-quality content and can cause confusion in the perception of “imagined” and “perceived” audiences (Goffman 1959), bringing to dangerous *sharing without caring* behaviors.
- **what**: what counts as *newsworthy* is not defined top-down by journalists, but it is frequently decided of popularity across social media; in such a way the prioritization of news flattens views which are not popular, fueling echo-chambers.
- **when**: a *news* is meant to constitute novel information at the moment of utterance; even though digital infrastructures allow for an almost “real time” access to events, they make it possible for a news to continue being circulated also when outdated and potentially overruled by following up events.
- **where**: the venues where a news spreads cannot be fully decided a priori by journalists or citizens: portions of a news are picked up and diffused through processes of reposting and re-sharing. This mechanism can bring to de-contextualization and cherry picking of information.
- **why**: the design of news feeds privileges claims in the form of clickbait news titles rather than editorial pieces. It does not come as a surprise that large-scale analysis of fact-checked news (Carmi, Musi and Aloumpi 2021) reveal that the lack of arguments supporting a news claim is a major indicator of misinformation. Studies in philosophy of education (Twardy 2004) have shown that teaching argumentation structures helps resilience toward unjustified claims.

Overall, the digital information ecosystem has enabled the formation of collaborative media (Lowgren and Reimer 2013): news production is a community effort of collection and interpretation of facts together with negotiation of opinions. In this *digital agora*, as in rhetorical arenas in Antiquity, persuasive communication strategies can be used both for enhancing information diffusion and for manipulation purposes. For the contemporary rhetorician, mastering digital skills is a prerequisite: an influencer and a fake news author both know how to leverage the digital environment to reach visibility and consensus. In this “likes” regime, what counts as newsworthy is not dissimilar from what turns out to be fake-newsworthy. Gaining critical thinking to become resilient to fake news entails becoming aware and questioning the impact of the aforementioned changes brought about by digitization.

## Human–computer interaction tools with gameful design to teach media literacy

It is now common ground knowledge that gameful design environments enhance learning due to a combination of unique features, such as visualization, immediate feedback, adaptation, challenge, competition, reward, fun failure (McGonigal 2001). These characteristics promote engagement, a driving force in the current information ecosystem: journalists need to engage communities in their everyday practices and account for social *media engagement* measures, such as public shares, likes and comments. Gameful design environments may, thus, help build users’ awareness about the very mechanisms which underlie engagement in their news feeds. Furthermore, Alt and Reichel (2018) show, through a longitudinal study, that problem solving is an asset to enhance digital skills advancing creativity. However, as pointed out by Luo (2021) in a comparative–contrastive study of 44 articles about educational gamification, effectiveness varies depending on several design factors which are not homogenous throughout gamification systems. To compare and contrast the suitability of different tools, we propose ten classification criteria and we then map how they are met by existing tools through the help of a focus group.

### Classification criteria

Our ten classification criteria encompass design and functionality to allow for comparison across tools.

(I) Explicit gameful design (McGonigal 2001): strategies that involve applications which are structurally game-like (Chou 2019); labels = (i) gamification (*gam*), elements from games are applied to a situation that is not a game (typical hallmark: badges); (ii) serious games (*sg*), games that have a specified ‘serious’ learning objective (iii) simulation (*sim*), activity that is designed to mimic a real-world scenario involving an interaction which does not necessarily entail gameplay;
(II) Implicit gameful design (Luo 2021): human-focused design techniques which affect the user experience without showing signs of a game; labels = (i) interaction (*inter*), the game focuses on how other agents, human or computer-controlled, interact with the player; (ii) achievement (*ach*), the design includes progression marks regardless the user’s performance (iii) competition (*com*), the design prompts the user to establish superiority over other players iv) challenge (*chal*): the design prompts the user to reach milestones in increasing levels of complexity v) fantasy (*fan*): the design posit the user in an imaginary scenario far in space and/or time from the real news-making process (vi) feedback (*fb*): the design allows for users to receive reactions from other game participants during their decision-making process rather than only afterward (vii) uncertainty (*unc*): the design does not allow the user to have expectations as to next steps.
(III) Pedagogical learning outcomes in relation to the **Five Ws described in Sect. 2:** labels = (i) *Who*, how to identify the role played by authors and audiences in (fake) news shaping and spreading? (ii) *What*, how to understand rationales behind the selection, presentation (headlines) and spread of certain topics? (iii) *Where*, how to investigate the role played by news venues in shaping information flows? (iv) *When*, how does the dimension of time affect (mis)information flow? (v) *Why*, how to assess whether a news claim is supported by sufficient and unflawed arguments?
(IV) Targeted type of media distortion: labels = disinformation (dis); misinformation (mis). Disinformation is intended as blatantly false information spread with the intention of causing harm, while misinformation is misleading information not necessarily crafted and shared with the intention of causing harm.
(V) Targeted type of media intervention: labels = debunking (de); pre-bunking (pre). Debunking is an intervention that “aims to retroactively reduce reliance on misinformation by correcting it once it has been encoded” (Tay et al. 2021), while pre-bunking proceeds in the opposite direction, reducing the persuasiveness of the misleading information before it is encoded. One of the major techniques employed for pre-bunking is *inoculation* which postulates that “by preemptively presenting someone with a weakened version of a misleading piece of information, a thought process is triggered that is analogous to the cultivation of ‘mental antibodies’, rendering the person immune to (undesirable) persuasion attempts” (Van der Linden and Roozenbeek 2020).
(VI) Perspectives selected to spot media distortions (fact-checked news): various aspects of the authors and the content of a news can be targeted in relation to media literacy; labels = source; factuality of news content; rhetorical strategies shaping news content
(VII) Number of human players: labels = one (1) or multiple (< 1) users at a time.
(VIII) Overall number of agents: labels = number of human players together with avatar(s) (1, < 2/2, < 3/3, 4/ < 4, 5/ < 5).
(IX) Dynamicity of scenarios: the news used in the gamification environment can vary as to actual occurrence and turn over; labels = (i) a fixed set of news that actually occurred (fixed) (ii) an automatically updated set of occurring news (updating) (iii) a made-up fixed set of news (made-up).
(X) Language: labels = language names (e.g., English, German) or multiple if more than two languages.

### A survey of human–computer interaction tools with gameful design for media literacy

To retrieve and analyze the tools available at the state-of-the-art, we have searched through *Google Scholar* for relevant academic papers using as filter keywords “game(ful)”/ “gamification” AND [mis/dis/information OR fake news OR digital/data/media/literacy].

We have then complemented the obtained list through the literature review contained in the collected papers. As a caveat, we have limited our list to tools containing explicit elements of gameful design in relation to data literacy. We, thus, excluded chatbots, such as IFCN's COVID-19 or Maldita.ed, which allow users to easily search for fact-checks on WhatsApp in Hindi and Spanish and get connected with local fact checkers through their smartphones. Though containing a human–computer interaction aspect, these tools are not gamified: they require users’ intentionality to discard fake news from their feeds rather than engaging them in a learning process for data literacy. We also did not consider tools such as *Test your argument* (Lawrence et al 2018 for an analysis), *DispuTool* (Haddadan et al. 2019) or which help the users recognizing the argumentative infrastructure of texts (standpoints, arguments etc.) exercising their critical thinking without, however, making direct reference to the (mis)information ecosystem. Furthermore, we had to eliminate from our survey *The News Hero* (https://thenewshero.org/) due to the presence of a broken link and *Allies and Aliens* (https://mediasmarts.ca/game/allies-and-aliens-mission-critical-thinking/kids) because it ran on Flash player which is not supported anymore.

It has to be noticed that our search excludes a plethora of games available online (mostly for mobile platforms) that deal with the topic of fake news outside the academic environment. Popular examples are *Fake news simulator* (https://play.google.com/store/apps/details?id=com.sangen.newswars&hl=it&gl=US), which provides the player with a set of randomized inputs that can be used to create humorous headlines, and *Idle Fake News Inc* (https://play.google.com/store/apps/details?id=br.com.tapps.fakenews&hl=it&gl=US), which nominally casts the player as the head of a disinformation website, but the gameplay remains that of a standard idle game. We did not consider them since they are not aimed at educating about disinformation, but at getting the player to create humorous or satirical news and share them making them popular without an evaluative stance.

Overall, we have identified 22 tools for media literacy with gameful design. We have played with all the tools apart from *FakeYou!* and *Incredible Times* which do not have public accessible interfaces but are respectively described in an academic paper (Clever et al 2020) and a webinar (https://media.edweb.net/edWebinar/?view=20180205edwebnet16).

We analyzed the characteristics of the tools based on our direct experience and the reports produced by the authors themselves. We then run a focus group composed of six doctoral students with mixed backgrounds (from communication to computer science) for 2 h: after being introduced to our categories, each student played with seven or eight tools (each toll has been analyzed by two students) and then noted the features they encountered. We then discussed the results focusing on cases of disagreement and then reached consensus. The most controversial category was *implicit gameful design* where “competition” and “challenge” were intended differently by different students since depending on the players’ priorities (overcoming others or personal progress); we thus refined our definition specifying that competition entails explicit mention to ‘others’ in the game design.

The results of the survey and the focus group give rise to the following taxonomy:

As shown in Table 1, the majority of games target disinformation (14/22), while 2 target misinformation only and the others blur the two categories. As to the type of intervention, a similar ratio is at stake with 14 tools with a pre-bunking function and 8 with a debunking one. In the *Vaccinating News* chatbot, for example, users are guided through a series of critical questions pointing to the potential presence of flawed arguments warning users to cast doubt on a piece of news (pre-bunking); on the other side in *NewWise*, for instance, their truth judgment over headlines is assessed through a direct debunk. Debunking tools mostly contain gamification elements, while prebunking is achieved with more sophisticated forms of explicit gameful design, such as simulations and serious games. The latter encourages engagement, instilling not only challenge and competition, but also achievement, uncertainty, fantasy and feedback. These implicit design features match with some of the 8 core principles of “flow theory” (Csikszentmihalyi 1993; Csikszentmihalyi and Csikszentmihalyi 1990), leveraged in game studies to guarantee players’ full immersion in the experience: feedback allows players to immediately know how well they are doing, setting up for clear goals; fantasy sponsors the “merging of action and awareness” as well as “concentration” and “loss of self-consciousness”; “achievement” guarantees that an adequate level of challenge is achieved without exceeding the player’s skills causing frustration; “uncertainty” fuels the “paradox of control” since it inhibits the player’s perceived control over the environment, while exercising his critical skills prompting inquisitiveness in a situation of potential risk.

**Table 1:** Classification of human–computer interaction tools with gameful design for data literacy

Gameful design tool	Explicit gd	Implicit gd	Learning outcome	(mis/dis)info	(pre/de)bunking	Fact-check trigger	*n* player	*n* agent	Type scenario	Lang
Bad News	sim, gam	inter ach unc fb	What Where	Dis	Pre	Content RS	1	2	Made-up	multiple
BBCiReporter	sim	inter chal	Who What Where	Mis Dis	Pre	Source Content RS	1	> 3	Fixed	English
Cranky Uncle	gam	ach fb	Why	Mis Dis	Pre	RS	1	2	Made-up	English
Factitious	gam	comp	What Where	Dis	De	Source	1	1	Fixed	English
Fakey	gam	comp	Where Who	Mis Dis	De	Source	1	1	Updating	English
FakeItToMakeIt	sg	chal	Who What When Where	Dis	Pre	RS	1	2	Made-up	English, German
FakeFinder	gam	chal	Who What Why	Dis	De	Source Content	1	> 3	Fixed	German
Fake News Detective	gam	chal	What Where	Dis	Pre	Source	1	2	Fixed	English
FakeNewsImmunity Chatbot	sg	inter fan fb	What Why	Mis	Pre	RS	1	5	Fixed	English
FakeYou!	sg	comp	What	Dis	De	Content	3	3	Updating	English
GoViral!	sim gam	inter ach unc fb	What Where	Dis	Pre	Content RS	1	2	Made-up	multiple
Incredible Times	sg	chal	Where When Who	Mis Dis	Pre	Source Content RS	1	2	Made-up	English
the Harmony Square	sg	inter chal ach unc	Who Where	Dis	Pre	Content RS	1	> 3	Made-up	English
MathE	sg	chal	What Why	Mis Dis	Pre	Source Content RS	1	1	Fixed	Greek
NewsWise	gam	chal fb	What	Dis	De	Content	1	1	Fixed	English
NewsFeed Defender	sg	comp	Who What When Why	Dis	Pre	Source Content	1	2	Made-up	English
Page1 Bingo	gam	chal	What	Dis	De	Content	1	1	Made-up	English
Real or Fake Photo Game	gam	chal	What	Dis	De	Content	1	1	Fixed	English
Real or Photoshop quiz by Adobe	gam	chal	What	Dis	De	Content	1	1	Fixed	English
The Evidence Toolkit	gam	chal fb	What Why	Mis Dis	Pre	RS	1	1	Fixed	English
Troll Factory	sim gam	inter chal ach unc	Who What Why	Dis	Pre	Content RS	1	2	Fixed	English
Vaccinating News Chatbot	sg	inter fan fb	What Why	Mis	Pre	RS	1	5	Fixed	English

The type of role play at stake is highly varied, ranging from that of an editor (*Incredible Times*) to that of a reporter (*BBCireporter*) or of a fake news spreader (*GoViral!*, *BadNews*). Even though more than one learning outcome can be achieved through a fact-check trigger at once, there is no gameful tool which covers all the proposed learning outcomes. *Troll Factory*, for instance, is one of the tools that addresses the largest number of learning outcomes, pointing to specific audiences to target (*who*), proposing different types of multimodal content to pick up (*what*), and explaining what is the rationale behind their strategic selection (*why*); however, no attention is devoted to the affordances offered by different social media platforms and the dimension of news timeliness. While more than half of the tools include multiple agents in the gameplay, only *FakeYou!* has multiple human players, while in the others all agents except the player are computer-controlled.

## Limitations of state-of-the-art gamification tools to tackle fake news

We identify several limitations of the surveyed gamification tools whose solutions represent a major challenge for the next developers. The first six paragraphs rely on the observations of patterns identified through the survey; they relate both to design and functional aspects. Section 4.7 rests on an aspect that goes beyond our taxonomy, pointing to the challenges imposed by the impact evaluation of digital tools with a gameful design to teach critical thinking for media literacy.

### Dangers connected with casting the player as the “bad guy” (criteria I, II)

Many tools (e.g., *Bad News*, *GoViral!* and *Fake It To Make It*) assign the player to the role of editor of a disinformation website. While this simulation is useful to understand the mechanisms behind the creation and diffusion of fake news, we should consider the risk of actually making the creators of fake news more sympathetic to the audience. From an implicit design perspective, this might favor a perception of *challenge* and maximize engagement. However, as shown by studies about video games design (Konijn et al. 2007), through a process of wishful identification (i.e., “what if I were the most viral spreader of fake news?”), players may develop empathy toward the characters they embody and perceive them as role models in their future behavior. To avoid this bias, *GoViral!* highlights face-threatening outcomes, showing to the player a text from a friend who is disgusted about his/her behavior. Going one step further, *Harmony Square* visualizes the harm caused by disinformation showing the neighborhood in which the game is set progressively degrading. Despite these countermeasures, fictional goals such as getting enough money to buy music equipment or pay rent, might make the decision to become a fake news spreader relatable and, thus, justifiable.

### No tangential learning (criterion III)

The educational purpose of these tools, despite its nature, is very clearly stated, thus attracting people who already have an interest in learning how to flag misinformation. To reach infodemically vulnerable people educational content about disinformation shall be incorporated in games with a broader scope.

### Scarcity of interventions specifically tackling misinformation (criterion IV)

**Fig. 1:**
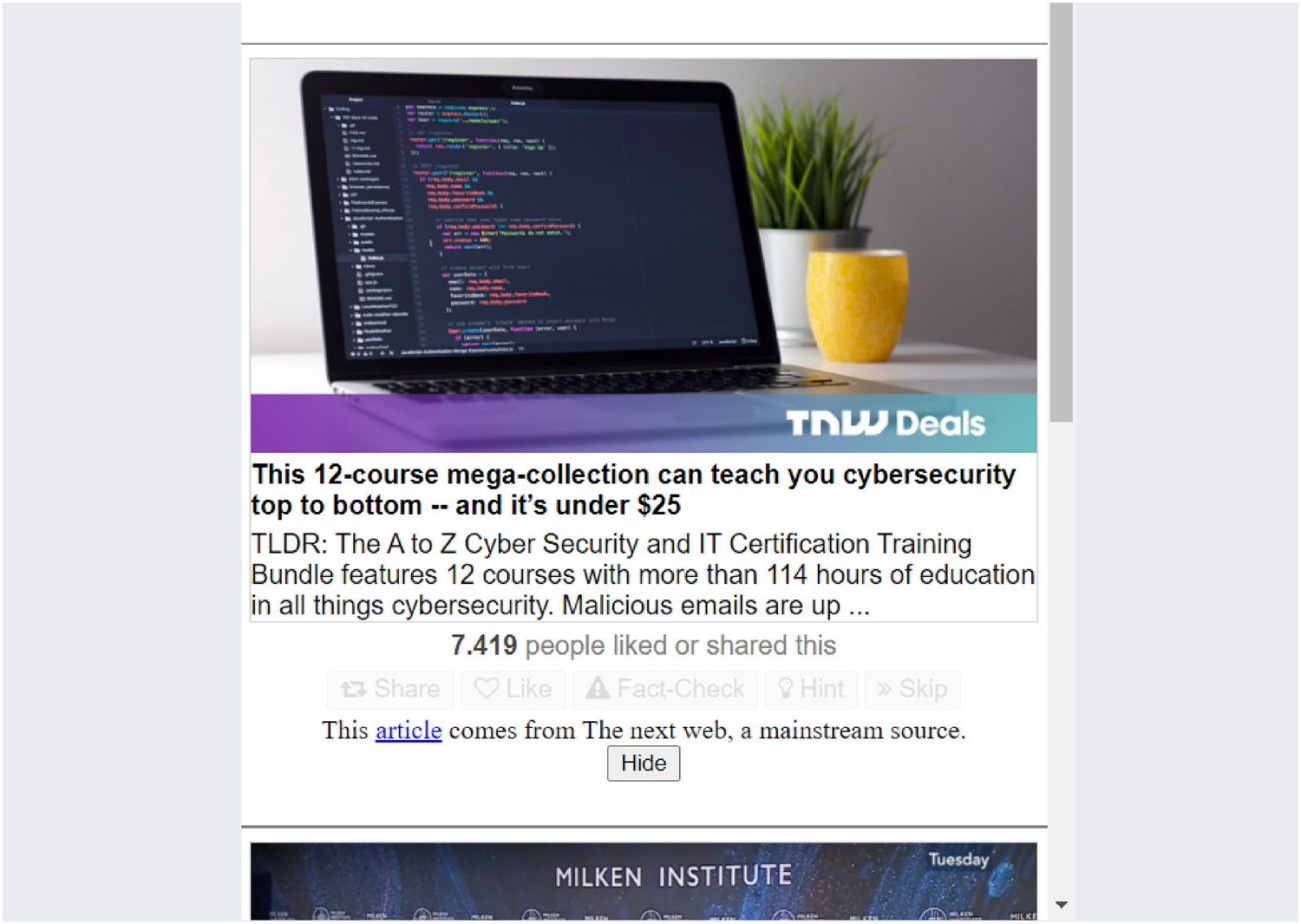
Advertisement of a cyber security course on “The next web”

While more than half of the reviewed tools (13) target disinformation, 2 tools only are devoted to counter specifically misinformation, and the other address both types of media distortions. The lack of focus on misinformation is, however, not justified: 59% of fake news present reconfigured rather than fabricated content (https://reutersinstitute.politics.ox.ac.uk/types-sources-and-claims-covid-19-misinformation), making misinformation at least as dangerous as disinformation. Furthermore, misinformation requires more training to be identified since un-intentionality makes, for instance, the author of the news a less relevant factor to disguise fake news (Fig. 1).

### Fact-checking instead of “argument-checking” (criteria V, VI)

Games that frame the source as the only proxy for the correctness of information are in danger of sending a message that is not of critical reasoning but of blindly trusting specific sources. *Fakey* is a particularly serious offender, as the game offers the player the option to pick one of three actions (like, share, fact-check) for every piece of news and when the player is presented news that the algorithm behind the game deems reliable (i.e., that come from mainstream sources) the decision to fact-check it is the one that gives the lowest score. Educational tools should always encourage the player to engage critically with content and cross-check multiple sources to determine what actually reliable news is.

A literal approach to fact-checking, that of checking adherence to the truth of the propositional content making up news, is in fact, not suitable to identify misinformation (Carmi et al. 2020): cherry picking of information can lead to highly misleading claims even if no blatantly false information is shared. In light of this, the tools in our survey which address misinformation next to disinformation focus on making aware about rhetorical strategies. However, tools countering disinformation primarily use debunking templates, instructing users to focus mostly on content and/or source. Such an approach is less promising since it does not educate users to be wary of (fake) content before being directly asked to do so. *NewsWise* or *Factitious*, for instance, show clickbait headlines and news to the player which is supposed to act as a fact-checker without, for example, being told that headlines sensationalized through sentiment are good candidates to be fake news. In such a way, the player, for the *continued influence effect* bias, will tend to believe the acquired information even after it has been corrected. As already pointed out in a study about climate change-related misinformation (Cook et al. 2017), explaining flawed argumentation techniques has a positive neutralizing effect against fake news and shall, we argue, be consistently extended to the fight of disinformation. Thus, in line with Visser et al. (2020), we believe that fact-checking should be supplemented with reason-checking, or more specifically, with “argument-checking” (Brave et al. 2022): it shall be evaluated whether the overall argumentative reasoning is acceptable, relevant, and sufficient. As argued by the creators of the term, “argument checking”, when applied to the digital sphere, not only has the advantage of empowering citizens to disentangling truth from fakey as individual users, but it also teaches individuals how to be better content producers.

In this regard, Plug and Wagemans (2020) propose a systematic procedure for “rhetoric checking”, through which argumentative content, structure and stylistic features are evaluated to bridge soundness of arguments to soundness of claims in the context of populistic discourse.

### Lack of social scenarios (criteria VII and VIII)

The current Networked Society has crucially shaped the information ecosystem, changing not only the way we access, but also how we consume, and react to news. Such a situation enables new participatory models of news production and a continuous ‘social’ negotiation of information where trust in peers plays a crucial role. To design effective training tools against misinformation it is, thus, important to account for such a dimension allowing multiple human players to negotiate the exchange of information next to artificial agents.

### Lack of reliably auto-updating content (criterion IX)

Some tools, especially quiz-like ones, front the user with specific examples of fake news accompanied by true reliable news. The proliferation of fake news makes static databases of examples become obsolete very quickly. Some tools use automated algorithms to search for new examples. At the moment, these systems are unable to detect fake news from the content and use proxies based on the reliability of the source. *Fakey*, for instance, scrapes news websites that have already been categorized as reliable or biased, assigning a truth value to the news on this basis. As a result, there is no fact-checking behind the cases presented and some content is mistakenly tagged as a reliable source of information just because it appears in a mainstream website even if it is not even a piece of news (e.g., an advertisement on a tech news website). The inclusion of automatically updated content is more challenging in the case of pre-bunking tools where content shall fit complex conversational human–computer interaction exchanges. Until now, none of the tools examined has tried to do so. One viable option would be that of guiding the user toward questioning news without offering a verdict, but providing links to debunking and fact-checking sites (and) image verification assistants.

### Vast majority of tools are in English language (criterion X)

Due to the academic origin of many of these tools, the vast majority of them are available in English only. The overwhelming majority of current scientific journals require the paper examining the structure of the tool and the effects of its deployment to be written in English, and this discourages researchers even from non-English-speaking countries from developing anything in other languages. *Bad News* and its new iterations *Go Viral!* and *Harmony Square* are translated in multiple languages, but the translation effort is crowdsourced and not centralized. As a result, *Bad News* is available in Esperanto but not in widely spoken languages, such as Spanish or Russian, thus significantly reducing the outreach.

### Un-systematic evaluation of impact

There is no unified framework to estimate the educational effectiveness of the tools to improve media literacy. Academic papers are available for 11 of the surveyed tools. We have to remark that with *evaluation* we do not refer to data retrieved from players’ interactions in relation to demographic features. As Grace and Hone (2019) pointed out, such data provide a useful snapshot of media literacy skills across the digitally included population, but they do not assess the impact that playing the game had in teaching critical thinking. The conclusions drawn from these reports are not conclusive and sometimes conflicting. Different proxies are in fact used in the evaluation: (i) *in game*: in-game tests that the player is asked to fill in before and after playing; gameplay statistics used to estimate how the skill level of the players changes the more they play; (ii) *out of game*: focus groups during which members are subject to elicitation tests after the game; feedback questionnaires that investigate whether media literacy skills have been acquired.

Starting from the latter, The *Fake News Immunity* and the *Vaccinating News Chatbot* provide a non-compulsory questionnaire in which players self-report their perceived increased ability of spotting fallacious news (93% positive) and are asked to explain the fallacy they have learnt. Similarly, Katsaounidou et al (2019), the developers of *Mathe,* designed an online field study with 111 participants to evaluate its impact and complemented the evaluation through a focus group with a media class (*n* = 35) discussing utilities and usability. The impact analysis of *Harmony Square* was carried (Roozenbeek and van der Linden 2020) over a test group of 681 participants, divided in a treatment group that played the game and a control group that played Tetris (Roozenbeek and van der Linden (2020) The experiment shows an improvement in the ability to spot disinformation, a decrease in the self-reported will to share it with friends and an increased confidence in their truth judgment. With regard to *Bad News* effectiveness, there are studies with opposing claims. Basol et al. (2021) identify an improvement in fake news distrust in a test group of 96 players after playing *Bad News*, compared to a control group of 102 subjects that played Tetris. Pimmer et al. (2020) claim that *Bad News* had less detectable impact over the ability of the players to identify fake news than the quiz-based approach of *FakeFinde*r, in which players are asked for a smart guess. Their study, however, is based on a small sample size (only 36 players for each tool). Furthermore, the group that played *Fake Finder* had significantly worse results in the pre-treatment test, while the two groups had very similar results in the final test. It is thus not clear whether the more significant improvement witnessed in the group that played *FakeFinder* is due to the greater effectiveness of the tool or to a ceiling effect. Basol et al. (2021) found that there is a minority of players for which playing *Bad News* actually increased the confidence in fake news, way more than for anybody else in the control group. This could be a result of the wishful identification effect we mentioned earlier, but further studies would be necessary to verify this justification.

Basol et al. (2021) assembled a focus group of 1777 players from the UK, France and Germany for the evaluation of *Go Viral!*. They divided the focus group into three test groups: a treatment group that played *GoViral!*, a control group that played Tetris and a third group that was given infographics about fake news detection to read. Also, three tests were taken, one before, one right after playing and one 1 week after the experiment. The results they obtained show significant differences between the outcomes of the second and the third tests. The test subjects who played *Go Viral!* showed a significant increase in skepticism toward both real and fake news right after playing, as a result of the over-sensibilization to the topic of fake news; after a week the distrust in real information went back to the original level while their increased ability to distrust fake news stayed. This result highlights at once the psychological effect of sensibilization to the danger of fake news and the possible distortion in the results of all tests taken right after the game inoculation. Though offering useful insights to improve the efficacy of the design, such methods do not shed light on long-lasting resilience across misinformation since they are non-longitudinal and often based on self-reported infos.

When it comes to *in-game* design, data are gathered automatically while the users are playing, either analyzing the gameplay and scores or from in-game questionnaires. The main issue is the lack of control by the authors on the composition of the test group. Furthermore, it is often difficult to reliably translate data about in-game behavior into information about data literacy, in particular for multiplayer games. Roozenbek and van der Linden (2019) indicated a positive impact of *Bad News*, using an in-game questionnaire taken by around 15,000 players. The authors, recognizing the limits of this approach, decided to follow up this paper with another one in which instead they assembled a focus group which also included a control group, which we have already mentioned (Basol et al. 2021). The creators of *FakeYou!* also included an evaluation of the effects it had on players (Clever et al. 2020), but, as remarked by the authors themselves, it is inconclusive due to the small sample size (53 players) and difficulty in decoupling the improvement of players in writing and recognizing fake headlines the more rounds they played. In general, for games with multiplayer competitive gameplay, in-game statistics are not reliable because they are always influenced by the skill level of the opponents. For *Fakey* an evaluation was carried out by the creators of the game (Micallef et al. 2021) of the in-game statistics of 8608 users over repeated sessions. The identities of players are tracked through social media login or cookies, which means that the identification of unique users might not be completely reliable. It shows that the players usually get more confident in mainstream media content the more rounds they play, but their faith in content from questionable websites also increases or at best stays the same. *The Evidence Toolkit* (Visser et al. 2020) allowed for users’ reviews that gave an overall positive evaluation of the app, which was found easy to use and able to get the users to analyze in greater depth the news.

## Conclusion

This study tackles the use of tools with gameful design to teach data literacy in a human computer-interaction environment to counter the infodemic. It provides a new classification framework to offer a theoretical and empirical roadmap to build next generation gameful design tools able to keep up with the fast spread of the infodemic. To achieve such goals we (i) define in operationalizable terms the blurred notion of data literacy, (ii) identify systematic and non-overlapping criteria to describe the design of the tools in relation to their educational goals, (iii) recognize challenges in developing tools which account for the complexity of the misinformation ecosystem and in measuring their effectiveness.

As to (i), we propose to conceptualize the current infosphere as an argumentative polylogue where multiple players negotiate multiple positions across different digital venues (social media, fora, newspapers) without a top–down gatekeeping process. Adopting this perspective, data literacy entails critical thinking not only of the news content (positions), but also data flows, platforms’ affordances and players’ motivations. We claim that the **Five Ws** generally used as a benchmark in investigative journalism have changed scope beyond the dimension of the single event: the same event is differently co-constructed across online communities leading to (mis)perceptions of addressed and imagined audiences (**who**), discrepancy between what’s timely in the real world and in the digital space (**when**), different criteria to assess newsworthiness (**what**), different chances to have a voice and justify that stance across different venues (**where** and **why**). In such a post-truth scenario, tools aimed at teaching critical thinking in the online media ecosystem shall address learning outcomes that encompass all these dimensions. The targeted learning outcome(s) constitute one of the ten criteria (ii) established to classify the tools, which encompass higher level features such as addressed type of media distortion and type of intervention (debunking vs pre-bunking), next to broad implicit/explicit gameful design features and more specific ones (fact-check trigger, language, number of players/agents; dynamicity of scenarios).

The survey, carried out through a keyword search on Google scholar, has surfaced 22 available gameful design tools for data literacy. Their comparative/contrastive analysis through our classification framework has revealed a set of seven main limitations which allow us to put forward corresponding recommendations to build more efficient gameful design tools:

- i**mplicit/explicit gameful design** better a**ligned with educational outcomes**: there are multiple tools and games that cast the player in a specific role related to (dis-mis)information (fact checker, spreader of disinformation, editor of a news outlet, etc.…). While each of these formats has advantages (e.g., evaluating critically the entire process of news creation and diffusion instead of only checking the final result) and risks (4.1., e.g., becoming empathetic with the “bad guy”), none of the games has multiple players interpreting different roles. A new approach could be that of having players interact as different figures in the online information diffusion world, using some form of asymmetric gameplay to challenge each other, as well as casting attention not only to the source and the content, but also the rhetorical strategies involved in spreading fake news. This situation would better mirror actual (mis)information flow dynamics which happen in a computer mediated communication environment and it could allow multiple learning outcomes at once.
- focus on **misinformation and prebunking**: the majority of the tools address disinformation only (4.2), while misinformation shall not be disregarded since news veridicality is often not black and white and calls for a skeptical, rather than judgmental eye. Prebunking interventions are preferable since they come along with implicit gameful design features that promote *flow* and, thus, engagement which prompts faster learning.
- promotion of **tangential learning**: all the tools are educational by design (4.3), while embedding educational content about disinformation in tools not explicitly educational would allow to reach an audience who is likely to be more vulnerable. To prompt engagement, an asset would be that of creating environments which trigger fantasy, diverting the attention of the user from the actual task toward an immersive storytelling. Furthemore, he comparison with non-academic fake news games sparse online suggests the possibility to insert humorous elements to balance gameplay and learning dynamic. For the sake of teaching media literacy, it would be fruitful to craft dialog and interaction templates which account for different types of humor: *relief humor* would contribute to relaxing face-threatening failures throughout the game, while *incongruity humor* would facilitate the acceptance of new perspectives reducing polarization (Meyer 2000).
- design of **tools to enable “argument-checking”** (Brave et al. 2022): the majority of the tools tackle one aspect of the (mis)information ecosystem, suggesting that facticity of content or credibility of sources are sufficient proxies to spot fake news (4.4). However, in the post-truth world, tools shall enhance epistemic vigilance focused on the analysis of the quantity and quality of arguments supporting a news claim.
- **reliable auto-updating content**: while the majority of tools present either made-up or fixed data cases (4.5), the few that leverage auto-updating content are not reliable. Since authentic problems are an important factor when teaching critical thinking (Abrami et al. 2015), more effort shall be paid to design accurate systems for outsourcing news content. The lack of automatic fact-checking tools also means that in many cases the authors of the tools used proxies for the actual truthfulness of the content, such as the authority of the news source. While this is hard to avoid when generating auto-updating content with currently available technology, it can negatively impact the educational value of the gameful tools encouraging blind trust in specific sources instead of critical thinking.
- **multi-language tools**: the majority of available tools are in English only (4.6), and those that are available in other languages are often made as translations of English language tools. Given the differences in the (dis-mis)information landscape across countries, it would be important to create native language tools, tailored to the specific characteristics of each environment.
- **framework for impact evaluation**: the major current challenge in the field of gamification to teach critical thinking is that of developing reliable evaluation metrics (4.7): both *in game* and *out of game* methods present some drawbacks. A possible way forwards is that of reformulating the evaluation of inoculation not as a measure of the performance in spotting fake news, but of the questions (type and number) that citizens develop when accessing news. The latter approach goes hand in hand with a conception of fake news immunity which rather than debunking or pre-bunking calls for the exercise of critical thinking.

Our analysis has some inherent limitations. First, the lack of a central official repository for educational tools means that, despite our best search efforts, we cannot consider our analyzed pool exhaustive of the educational tools with gameful design created by academic researchers to contrast dis-mis-information. Further, we played these games just as users would, with no access to the source code and no developer mode enabled in-game. We explored the content of each tool in as much detail as possible, but this means that, in particular for games with branching stories, we could have missed some events and functionalities. Finally, for the evaluation of the impact, we relied on the data provided by the authors in academic papers accompanying the tools and did not run our own evaluations assembling our own focus group to get more homogeneous data.

The review of state-of-the-art educational tools with gameful design to tackle dis-misinformation highlights the need for further work not only to develop new tools, but to shape a general research framework around them. We recognize the need for a unified framework to evaluate the impact of these tools, following standardized procedures, to reliably estimate and compare their educational effect. In particular, there should be control over the composition of the test groups and uniformity as to control groups the users are tested against, time distribution of tests and questions that are asked in the questionnaires. As to the latter, we believe that the *California Critical Thinking Skills* Test (CCTST) developed by Peter Facione (1990, 1992) provides conceptual backbones. Its main principles (interpretation, analysis, evaluation, explanation and inference) are leveraged by the Intelligence Community (IC) Rating Scale for Evaluating Analytic Tradecraft Standards (ODN 2015) and the so-called sense-making scale developed by Alsufiani et al. (2017). Adaptations of both scales have been used to successfully elaborate collaborative intelligence tools (van Gelder et al 2020; De Liddo et al. 2021). It will, however, be necessary to attune these scales to the (mis)information ecosystem. A major challenge for the evaluation of the impact of multiplayer games will be finding measures to account for how the actions of other players influence the learning outcomes. Furthermore, phenomena such as the danger of wishful identification with the “bad guys” call for closer examination. The landscape of the dis-misinformation phenomena and of the efforts to tackle them is constantly evolving and thus the aggregation, analysis and classification of the tools developed has to be regularly updated.

## Footnotes

1 Although the whole paper has been the result of a continuous process of interaction among the three authors, Elena Musi is the main responsible of Sects. 1, 2, 3.1 and 5, while Lorenzo Federico of Sects. 3.2 and 4; Gianni Riotta has contributed to the survey analysis and the elaboration of recommendations.
